# Influence of Chitosan Extraction Process from Invasive Crayfish (*Faxonius limosus*) Shells on Properties Relevant to Active Food Coatings

**DOI:** 10.3390/gels12080664

**Published:** 2026-07-24

**Authors:** Nevena Hromiš, Senka Popović, Zorica Tomičić, Nadežda Seratlić, Danijela Šuput, Jovana Pantić, Ivana Čabarkapa

**Affiliations:** 1 Faculty of Technology Novi Sad, University of Novi Sad, Bulevar cara Lazara 1, 21000 Novi Sad, Serbia; madjarev@uns.ac.rs (S.P.); danijela.pejic@uns.ac.rs (D.Š.); jovana.ugarkovic@uns.ac.rs (J.P.); 2 Institute of Food Technology, University of Novi Sad, Bulevar cara Lazara 1, 21000 Novi Sad, Serbia; zorica.tomicic@fins.uns.ac.rs (Z.T.); nadezda.seratlic@fins.uns.ac.rs (N.S.); ivana.cabarkapa@fins.uns.ac.rs (I.Č.)

**Keywords:** *Faxonius limosus*, shell waste, viscoelastic chitosan polymer network, edible coating–forming matrices, fruit, rheological properties, wettability, antimicrobial activity, antioxidant activity

## Abstract

To control the impact of the invasive crayfish *Faxonius limosus* on native crayfish and fish biodiversity in the Danube River ecosystem, one possible approach is the valorization of this species through the production of value-added biopolymers, considering the continuously increasing demand for chitosan. However, there are very limited data regarding the utilization of *Faxonius limosus* shell waste as a source of chitosan. Therefore, this study evaluated chitosan recovery from spiny-cheek crayfish shell, including conventional chemical treatment with different demineralization intensities and numbers of deproteinization steps, as well as ultrasound and autolysis-assisted deproteinization. The obtained chitosans were characterized in terms of yield, moisture content, degree of deacetylation, color, crystallinity and structural properties. Residual heavy metal concentrations (Hg, Cd and Pb) were determined to assess the safety of crayfish shell as a raw material intended for food-related applications. Particular emphasis was placed on gel-related functional properties of obtained chitosans, including rheological behavior, wettability on fruit surfaces, antioxidant and antimicrobial activities, and film-forming ability. These properties govern the formation of structured biopolymeric networks and their performance as active food coating materials. The relationships between the extraction process, physicochemical characteristics and functional performance were investigated to identify the most suitable chitosan for potential food preservation applications. The results demonstrated that extraction conditions significantly affected the physicochemical and functional properties of chitosan. Samples obtained through intensive deproteinization showed enhanced antimicrobial activity, whereas higher antioxidant activity was observed in samples containing residual bioactive compounds. Most formulations exhibited suitable wettability on apple and nectarine surfaces and successfully formed transparent films, indicating their potential application as edible coatings.

## 1. Introduction

*Faxonius limosus* (formerly *Orconectes limosus*) is one of the most invasive freshwater crayfish species in Europe and is now widely distributed in the Danube basin. The species originates from North America and was deliberately introduced to Europe in Germany in 1890, from where it gradually spread through the river systems of central and Eastern Europe. After entering the Danube system, populations were recorded in Germany, Austria, Hungary, Croatia, Serbia, Romania and Bulgaria [1,2,3,4].

In Serbia, the species was first recorded in the early 2000s and is now present along almost the entire course of the Danube through Serbia, as well as in certain tributaries. The DANUBEcare project reports that the species has “established itself along the entire Serbian sector of the Danube and its tributaries”, with further expansion expected [5]. Research by Zorić et al. (2020) identified seven new localities on the main course of the Danube, with the authors concluding that colonization occurs in both directions along the river corridor and that the species continues to actively spread [2]. Roljić et al. (2024) state that *F. limosus* is one of the most invasive alien species in Europe and confirm stable populations in the Serbian part of the Danube approximately two decades after the first record [6].

The Invasive potential of the species Is based on several biological characteristics, such as high reproductive capacity, rapid maturation, broad ecological tolerance, the ability to colonize various habitats, and resistance to adverse environmental conditions. Additionally, the species is a carrier of the causative agent of crayfish plague (*Aphanomyces astaci*), to which European native species are highly susceptible. Consequently, invasive populations are linked to the decline of native species such as *Astacus astacus* and *Pontastacus leptodactylus*. Besides transmitting pathogens, the species competes with native crustaceans for shelter and food, alters the structure of benthic communities, and affects trophic relationships within the ecosystem [1,7,8]. Since 2016, it has been included in the List of Invasive Alien Species of Union Concern for the European Union [4].

Consequently, population control and removal programs are widely recommended as part of invasive species management strategies. Converting crayfish shell waste into value-added chitosan is consistent with circular bioeconomy principles and may contribute to reducing the environmental burden associated with invasive species control. Reducing the negative impact of this invasive species on the biodiversity of the Danube could be achieved through smart exploitation. Vidosavljević et al. (2025) and Lazarevic et al. (2022) have already reported that *F. limosus* meat is a nutritionally valuable and safe alternative protein source [9,10]. After utilizing the crayfish meat, it is possible to develop high-value-added products based on chitin and chitosan from the crayfish shell. For countries such as Serbia, which lack access to the sea and do not have developed sea crustacean processing, crayfish shells generated as by-products of crayfish utilization for human consumption or animal feed may represent an important local source of biomass from both economic and ecological perspectives. In this context, the exploitation of invasive freshwater crayfish for valuable food and feed products leaves the shell as a potentially significant source of chitin and chitosan for continental European countries [11,12].

The literature on the isolation of chitin and chitosan from the shell of the crayfish *F. limosus* is very limited. The only published work on this topic is by Nuc et al. (2023), who studied two processes for extracting chitin and chitosan from the shell of *F. limosus*: a conventional chemical method and extraction using natural deep eutectic solvent (NADES) mixtures [13]. They reported yields, structural and thermal properties, antimicrobial activity, and cytotoxicity of the isolated chitosans. To advance research in this area, this study applied the chemical process of chitosan extraction with various modifications to potentially shorten and simplify the process, and to reduce chemical usage and wastewater production. Chitosans obtained from different modifications of the chemical process were subjected to chemical, structural and bioactivity analyses, but the scope of analysis was extended to include application properties related to the behavior of the resulting chitosan solutions for use as coatings and films. Chitosan solutions are widely used as hydrogel precursor systems due to their ability to form structured polymeric networks through physical or chemical interactions. The rheological behavior and wettability of such systems are important parameters governing their subsequent application as hydrogels, films and edible coatings. Therefore, the aim of this study was not only to valorize shells of the invasive crayfish *Faxonius limosus*, but also to evaluate how different extraction processes affect the physicochemical, and gel-related functional properties of chitosan, including rheological behavior, polymer network-forming potential, antioxidant and antimicrobial activities, film-forming ability and wettability on fruit surfaces, which are critical for testing its potential for edible coating application.

## 2. Results and Discussion

### 2.1. Yield

The yield of chitosan from different processes ranged from 9.42 ± 0.9% and 10.15 ± 1.02% for samples 0DP and 3DP, to 13.81% and 13.82% for samples UZ and A in the series demineralized to pH 1.3 (DM1.3 series) (Table 1). In the series demineralized to pH 0.5 (DM0.5 series), these values ranged from 6.27 ± 0.63% and 6.57 ± 0.66% for 3DP2 and K, to 8.19 ± 0.82% and 9.41 ± 0.94% for UZ2 and 0DP2. Two-way ANOVA showed that the demineralization series significantly affected chitosan yield (F = 42.60, *p* < 0.001, η^2^ = 0.559), whereas the extraction treatment (F = 2.82, *p* = 0.066, η^2^ = 0.185) and the interaction between the two factors (F = 1.51, *p* = 0.260, η^2^ = 0.099) were not significant.

The yield of the pre-deacetylation material decreased with increasing treatment intensity. In the first series, demineralized material yield was 43.00% for 0DP, while the yield after one, two and three deproteinization steps decreased to 26.78, 21.08 and 14.74%, respectively. A similar trend was observed in the second series, where the corresponding values decreased from 37.00% for 0DP2 to 21.52, 16.36 and 15.00% for 1DP2, 2DP2 and 3DP2, respectively. The generally lower yields in the DM0.5 series may be associated with the more intensive demineralization to pH 0.5 compared with pH 1.3 in the DM1.3 series.

### 2.2. The Moisture Content

The moisture content of the tested samples ranged from 4.5% to 7.5%. The DM1.3 series of samples had moisture content close to 4.5%, while samples from the DM0.5 series, subjected to several purification steps, showed moisture contents above 6% and 7%. Two-way ANOVA showed that moisture content was significantly affected by the demineralization series (F = 329.2, *p* < 0.001, η^2^ = 0.600), extraction treatment (F = 20.2, *p* < 0.001, η^2^ = 0.184), and their interaction (F = 21.3, *p* < 0.001, η^2^ = 0.194), indicating that the effect of the extraction treatment on moisture content depended on the demineralization conditions. More intensive demineralization and deproteinization may expose a greater number of polar amino and hydroxyl groups, increasing the affinity of chitosan for water and consequently its moisture content.

### 2.3. The Degree of Deacetylation

The degree of deacetylation of the analyzed chitosan samples was within the narrow range of 70–75%. Two-way ANOVA showed that neither the demineralization series (*p* = 0.947), extraction treatment (*p* = 0.998), nor their interaction (*p* = 0.997) had a significant effect on the degree of deacetylation.

### 2.4. Total Amino Acid Composition

Total amino acid composition was analyzed for DM1.3 samples to test and monitor different deproteinization techniques. Results in Table 2 showed that total amino acid content was relatively low for all tested samples, suggesting that the deacetylation process influences simultaneous deproteinization, as observed in sample 0DP. However, multiple deproteinization steps led to a further decrease in amino acid, and thus protein, content in the tested samples.

**Figure 1:**
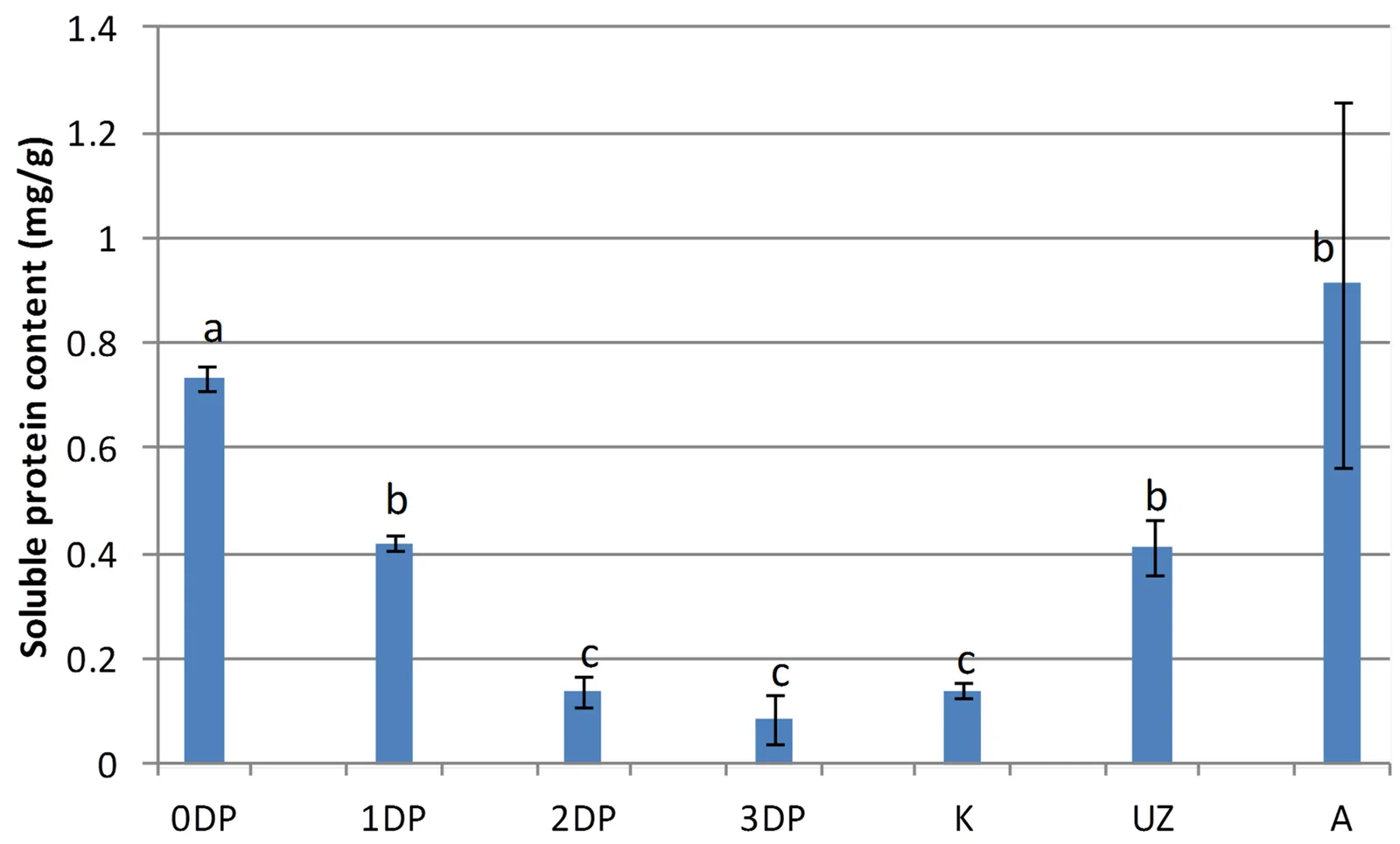
Soluble protein content in the analyzed samples of series demineralized to pH 1.3 (different letters a, b, c denote significantly different values).

In addition to amino acid content in the tested samples, soluble protein content in the analyzed samples was measured using the Lowry method. The results also confirm a decrease in protein content with an increased number of deproteinization steps (Figure 1).

### 2.5. Color Parameters of Chitosan Powder Samples

**Figure 2:**
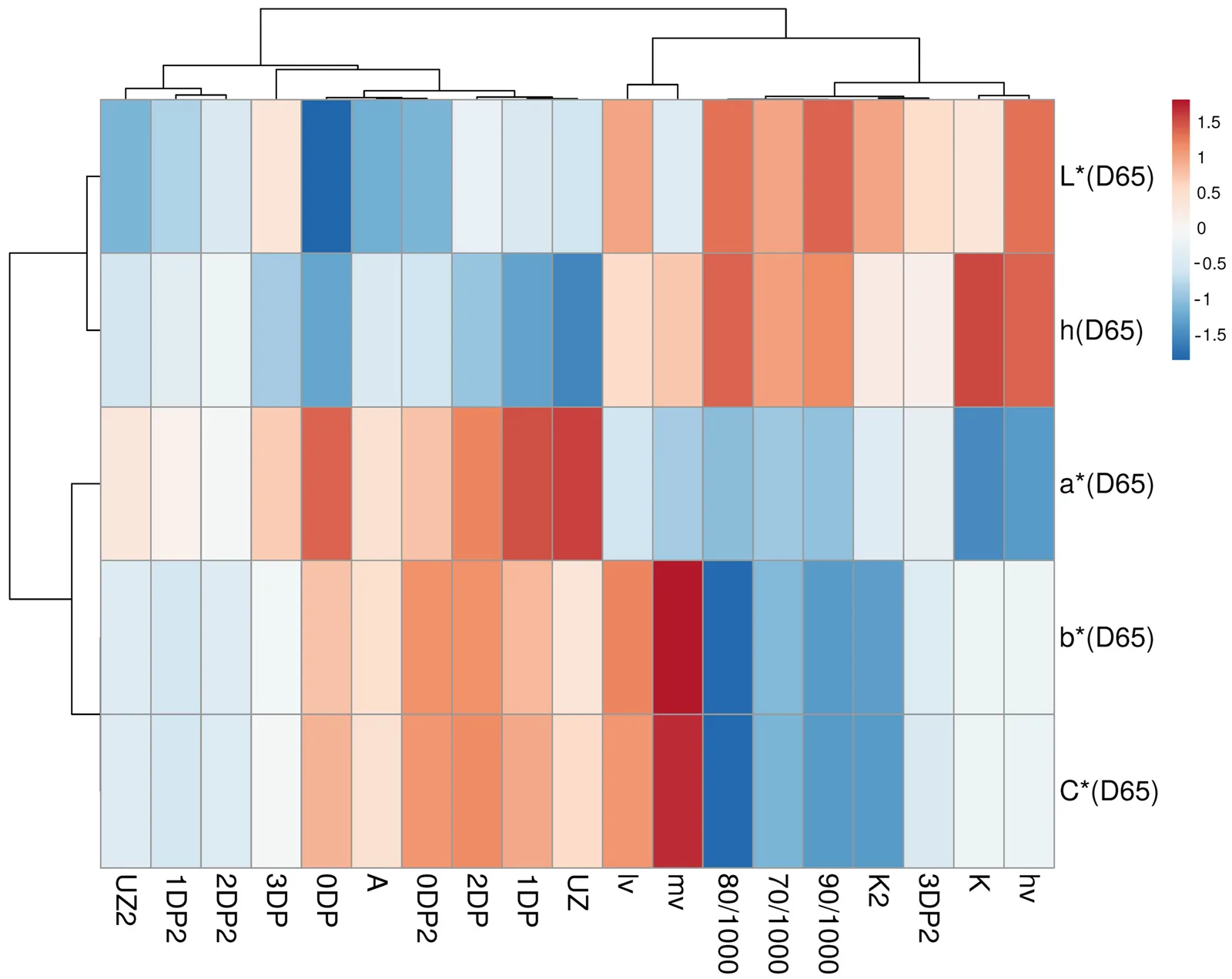
The heat map and cluster analysis of color parameters of different chitosan samples. L*, a*, b*, C* and h denote the CIELAB color parameters: lightness, green–red coordinate, blue–yellow coordinate, chroma, and hue angle, respectively.The dendrograms represent the hierarchical clustering of the samples (top) and color parameters (left), based on their similarity.

Based on the heatmap dendrogram of color parameters of chitosan powder samples (Figure 2), the primary separation goes into two clusters (the highest two branches): the left, containing all the laboratory samples, and the right, which includes six commercial samples and three laboratory samples (K, K2, and 3DP2). The laboratory samples in the left branch are more similar to each other than the commercial samples and the three laboratory samples in the right branch are more similar to each other. Within the left branch, there are three groups: sample 3DP stands alone; the second group (cluster) consists of 0DP, A, 0DP2, 1DP, 2DP, and UZ; and the third group (cluster) comprises UZ2, 1DP2, and 2DP2. Clustering can be further subdivided, but this is the basic trend.

The heatmap and cluster analysis of color parameters show that samples from the group 0DP, A, 0DP2, 2DP, 1DP, and UZ (mainly DM 1.3 sample series and those with milder treatments) have the lowest brightness and the highest chromaticity, with pronounced orange-yellow tones. Group 4 (commercial samples and K, K2, and 3DP2) is characterized by the highest brightness, weak chromaticity, and shades close to white. The groups UZ2, 1DP2, and 2DP2 (mainly the DM 0.5 sample series) have cooler tones and moderate lightness, while 3DP is isolated as a sample with medium brightness and moderately warm shades.

High brightness and an appearance close to commercial samples were achieved with both K samples, but also with sample 3DP2, which was not additionally depigmented, significantly saving time and consumption of chemicals. Low chromaticity and medium lightness were achieved with sample 3DP, as well as with samples of the DM0.5 series, while the other samples show an obvious residue of colored components.

### 2.6. The Appearance of Chitosans

**Figure 3:**
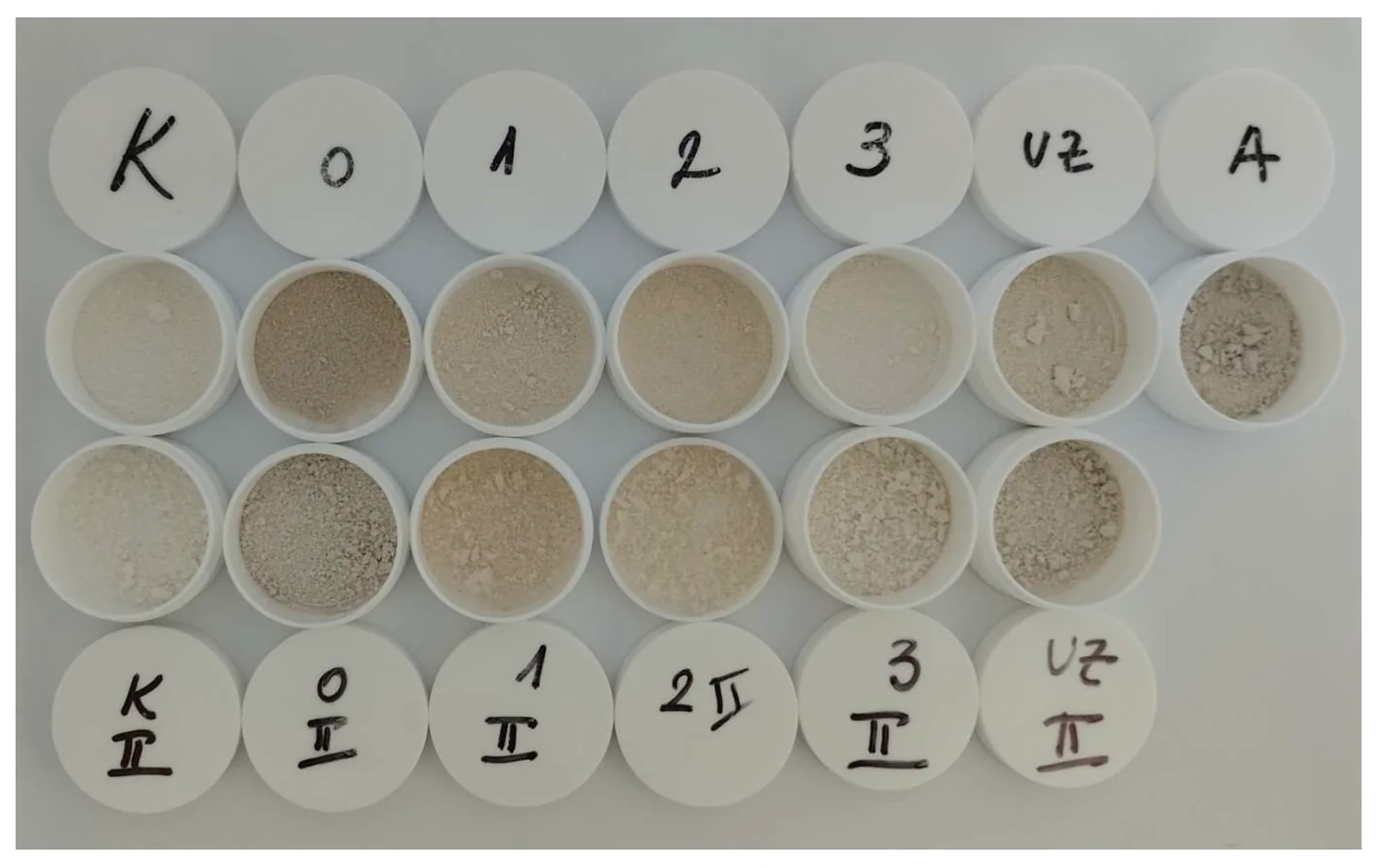
Visual appearance of obtained chitosans. 0, 1, 2 and 3 represent samples demineralized to pH 1.3 and subjected to 0, 1, 2 or 3 deproteinization baths, respectively. K denotes the sample additionally subjected to depigmentation, A the autolysis-treated sample, and UZ the ultrasound-assisted deproteinized sample. Samples marked with the suffix “II” (0II, 1II, 2II, 3II, KII and UZII) were demineralized to pH 0.5 before the corresponding deproteinization treatment.

The appearance of chitosans obtained by different treatments is shown in the photo (Figure 3). The commercial chitosans are whitish, as are the 3DP and K samples of both series, while the others have more or less reddish-yellowish or gray-brownish coloration, which most likely indicates residual pigments, protein and/or non-ferrous metals.

### 2.7. The Metal Content

**Figure 4:**
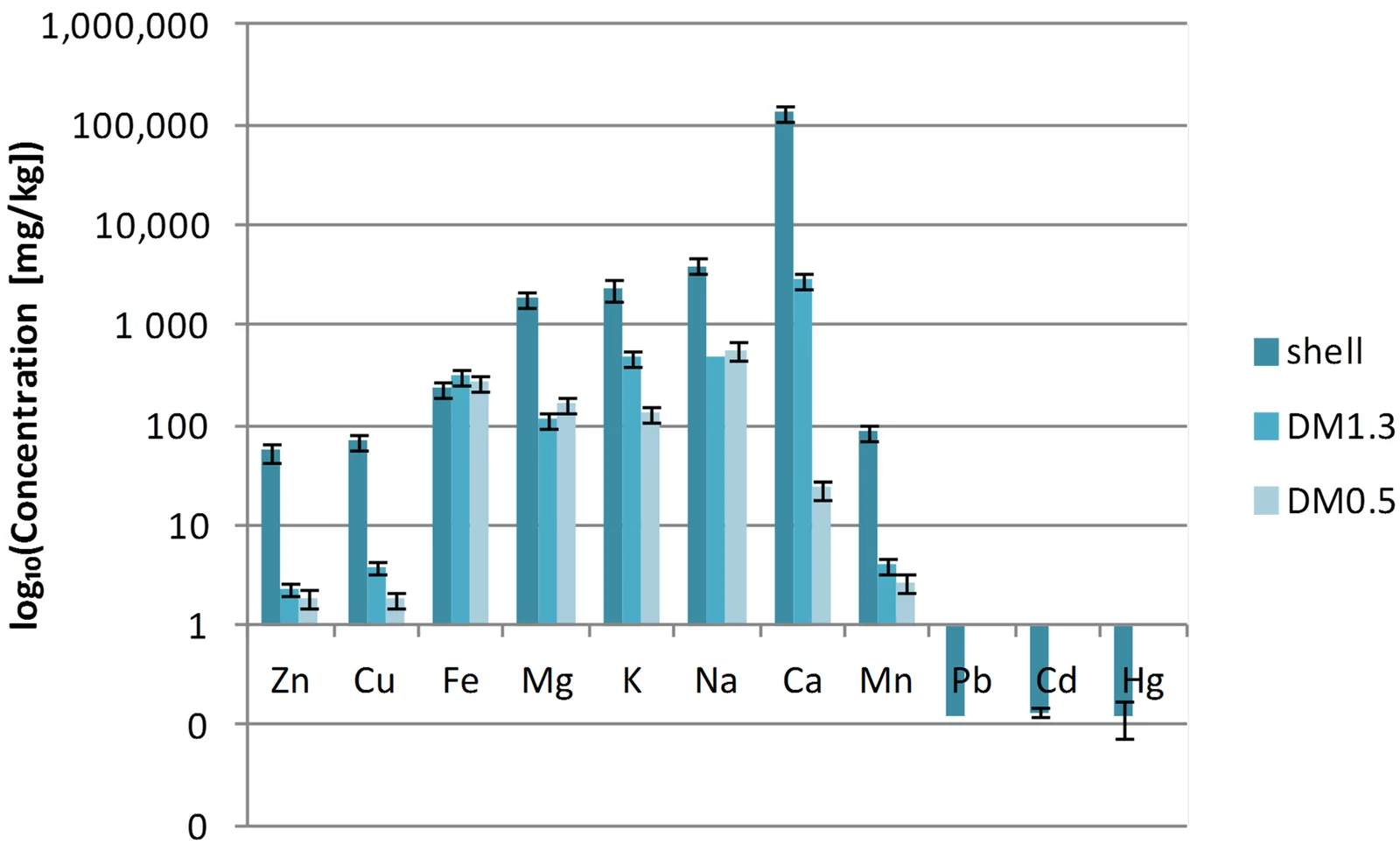
Metal content in the crayfish shell, chitin DM1.3 and DM0.5. The results are presented on a logarithmic scale.

Figure 4 shows the metal content in the crayfish shell, as well as in chitin DM1.3 and DM0.5. Both chitin samples were deproteinized in one step. These samples are representative of chitosans 1DP and 1DP2. The results are presented on a logarithmic scale because the content of individual metals varies greatly between samples. Heavy metal content in the shell was 0.183 ± 0.06 mg/kg for Pb, 0.136 ± 0.05 mg/kg for Cd and 0.124 ± 0.033 mg/kg for Hg, all below the permitted level for crustaceans (meaning crustacean meat) in Commission Regulation (EU) 2023/915, where the maximum permitted level for these metals is 0.5 mg/kg for Pb, Cd and Hg [14]. Since the concentrations of Pb, Cd and Hg in the raw shells were already below the maximum limits established for food applications, and no heavy metal-containing reagents were introduced during the subsequent deproteinization and deacetylation steps, an increase in heavy metal concentration in the final chitosan is not expected. The other metal content in the shell was very high for most metals. By far the largest proportion of all metals is Ca, followed by Na, K, Mg, then Fe, and then Mn, Zn and Cu. In chitin DM1.3, the content of all metals was significantly reduced compared to the shell (Ca content is at 2% of the shell content). The demineralization process was effective. However, in the DM0.5 chitin sample, the metal content was further reduced (Ca content is at 0.02% of the shell Ca content). It can be observed that the iron content in both tested series remains high (minimally reduced compared to the shell), which can significantly affect the color of the obtained chitosan (yellow, ochre, and orange to brown color of Fe^3+^ ions, with possible complexation and oxidation resulting in a greenish or reddish hue). In the presence of pigments, these shades can be intensified.

### 2.8. FTIR Spectra Analysis

**Figure 5:**
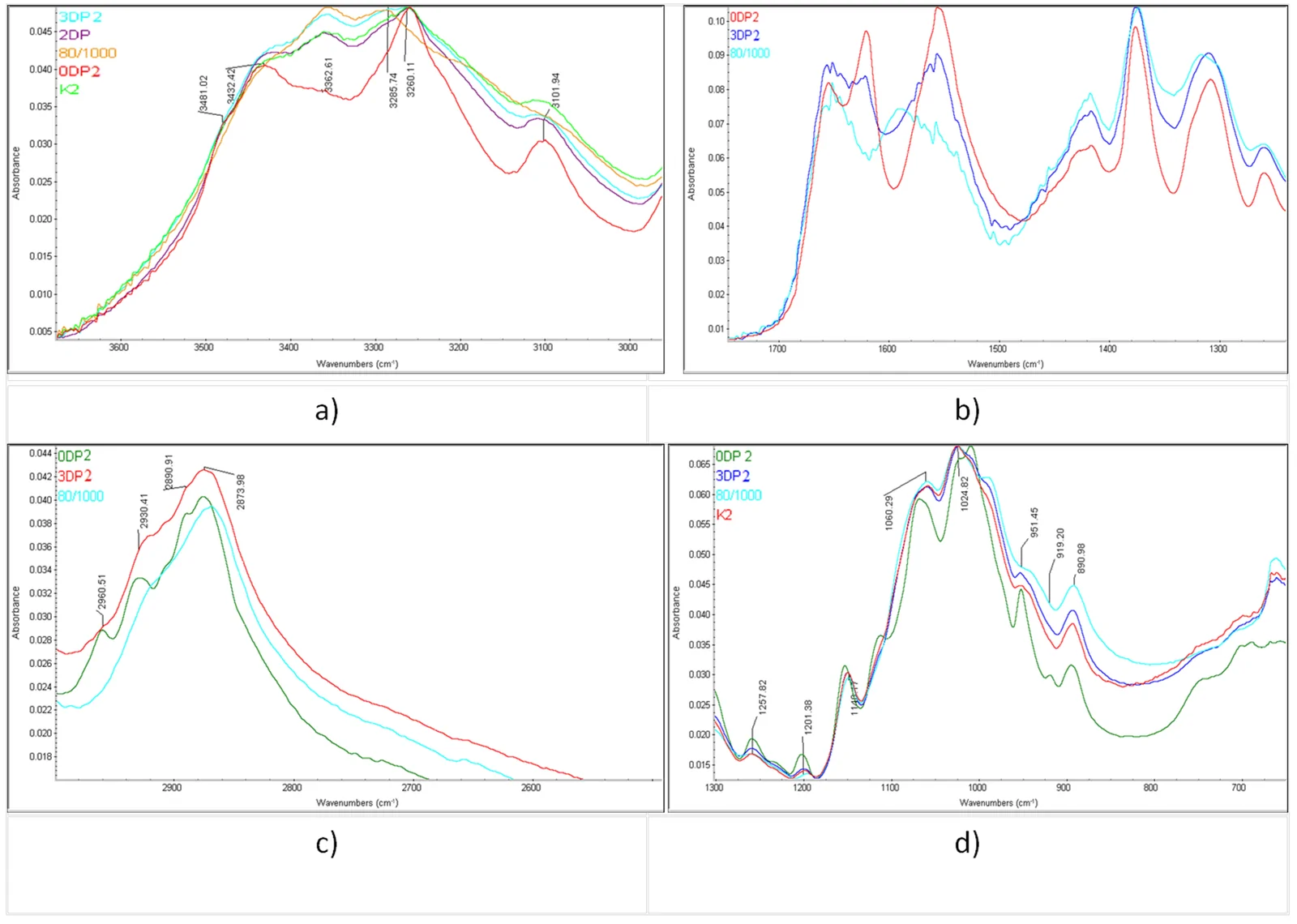
FTIR spectra sections of commercial sample 80/1000 and experimental samples: (**a**) Spectral range 3600–3000 cm^−1^. (**b**) Spectral range 1700–1300 cm^−1^. (**c**) Spectral range 3000–2500 cm^−1^. (**d**) Spectral range 1300–600 cm^−1^.

FTIR spectra analysis in Figure 5 shows certain differences in the region 3000–3600 cm^−1^. The two extreme forms are 0DP2 (with which UZ2 and A are very similar) and 3DP2 (with which K2 and 2DP2 are similar). Other spectra fall between these two groups in terms of peak intensity and position. Peaks in this area are related to vibrations crucial for the analysis of –OH and –NH groups, the presence of free water, and intermolecular hydrogen bonds. In the region around 3350–3600 cm^−1^, where the –OH stretching vibration band from the molecule and from moisture is located, experimental samples show a slightly higher proportion of these bonds compared to the commercial standard sample. The section concerning the N–H bonds in the chitosan molecule, which also provides information about possibly present residual proteins, differs between commercial and experimental samples. In the commercial sample, this peak is located at 3285.74 cm^−1^, while in the experimental samples it is shifted to 3260.11 cm^−1^ and is of increased intensity, rising from samples 3DP2, K2, and 2DP2 to 0DP2, UZ2, and A, with the other samples falling in between. An increase in intensity and a shift to lower wavenumbers indicate the presence of residual proteins in the samples. This is supported by the peak at 3101.94 cm^−1^, which follows the same trend. The region of the spectrum 3000–3600 cm^−1^, observed together with the part of the spectrum describing the amide I (1700–1600 cm^−1^), amide II (1600–1500 cm^−1^) and amide III (1315–1320 cm^−1^) groups reveals the higher intensity peaks shift to lower wavenumbers compared to the commercial standard sample, confirming the presence of proteins in the samples in increasing order from 3DP2, K2, and 2DP2 to 0DP2, UZ2, and A, while the other samples fall between these [15,16,17]. This interpretation is in agreement with the independent determination of total amino acids and soluble proteins in representative chitosan samples, which showed a progressive reduction in proteinaceous material with increasing deproteinization intensity.

C-H groups from chitosan are observed as a band at 2870 cm^−1^ in the commercial sample. The band broadens towards 2960 cm^−1^ due to the presence of various C-H bonds in chitosan. Sample 3DP2, which is most similar to the commercial sample, shows a comparable appearance of this peak, while sample 0DP2 displays additional peaks likely originating from nonpolar lipid impurities or protein residues. The remaining experimental samples, based on the appearance of the spectrum in this region, fall between these two spectra. Similarly, in the 600–1300 cm^−1^ region, where peaks originate from the chain skeleton of chitosan molecules (-C-O-C- bridges), peaks at 1148 cm^−1^, 1060 cm^−1^, 1025 cm^−1^, and 891 cm^−1^ are observed. The presence of more pronounced and new peaks indicates organic impurities. The FTIR analysis results are consistent with those obtained from instrumental color determination, amino acid and protein content.

### 2.9. X-Ray Crystallography

**Figure 6:**
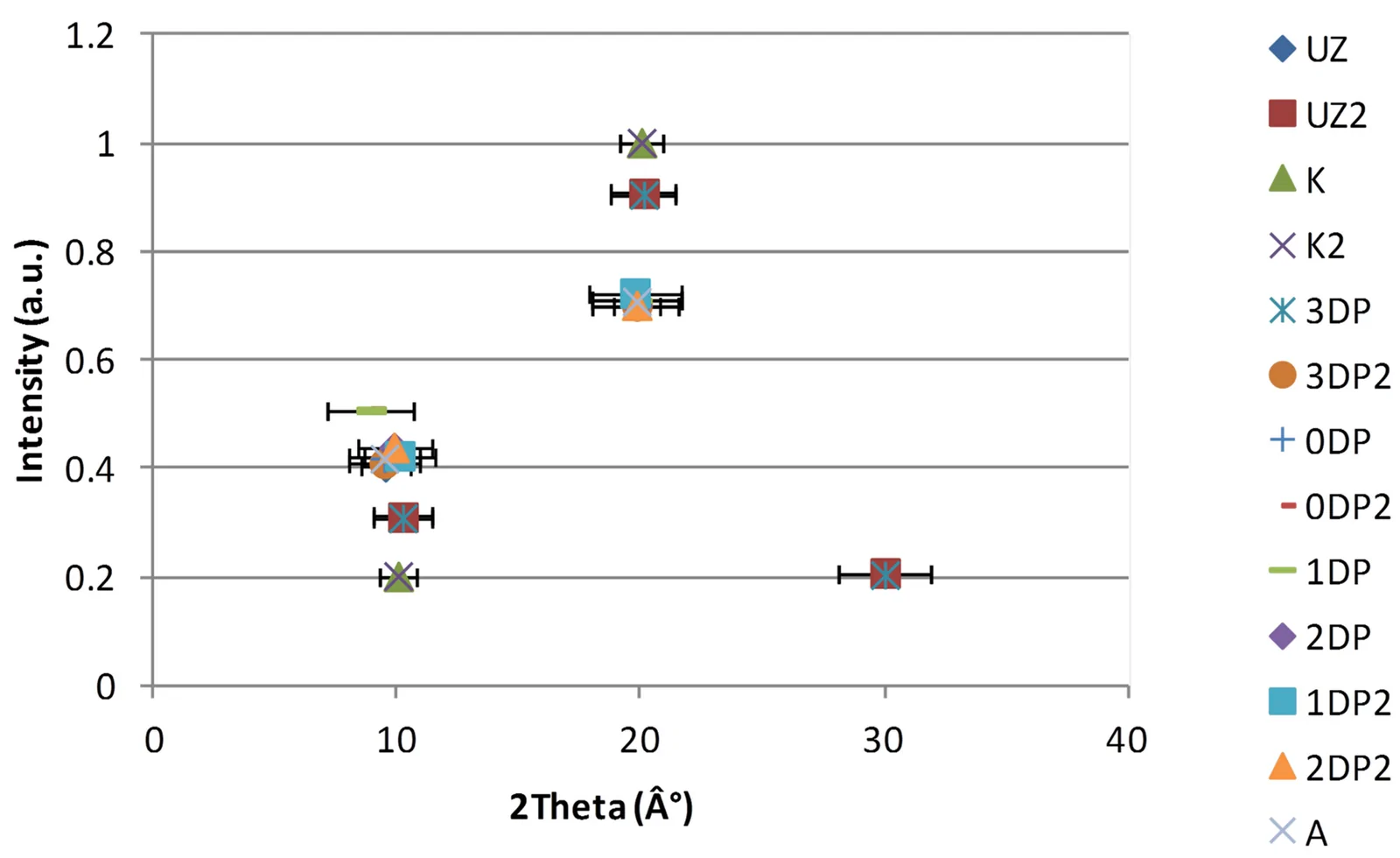
The shape of the XRD crystallographic peaks of all tested samples; the point represents the maximum, and the error bar represents the width of the peak.

**Figure A1:**
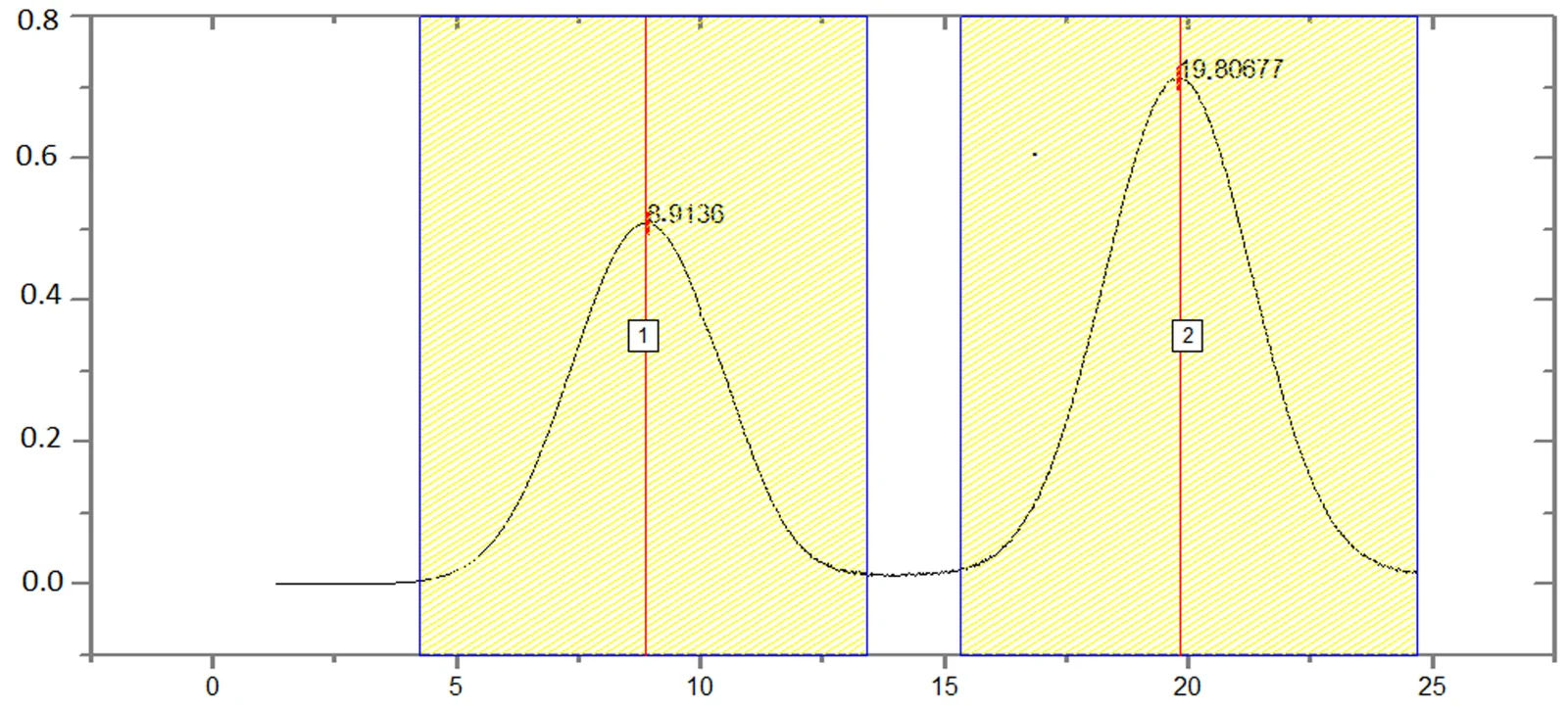
XRD crystallographic peaks for 0DP2.

**Figure A2:**
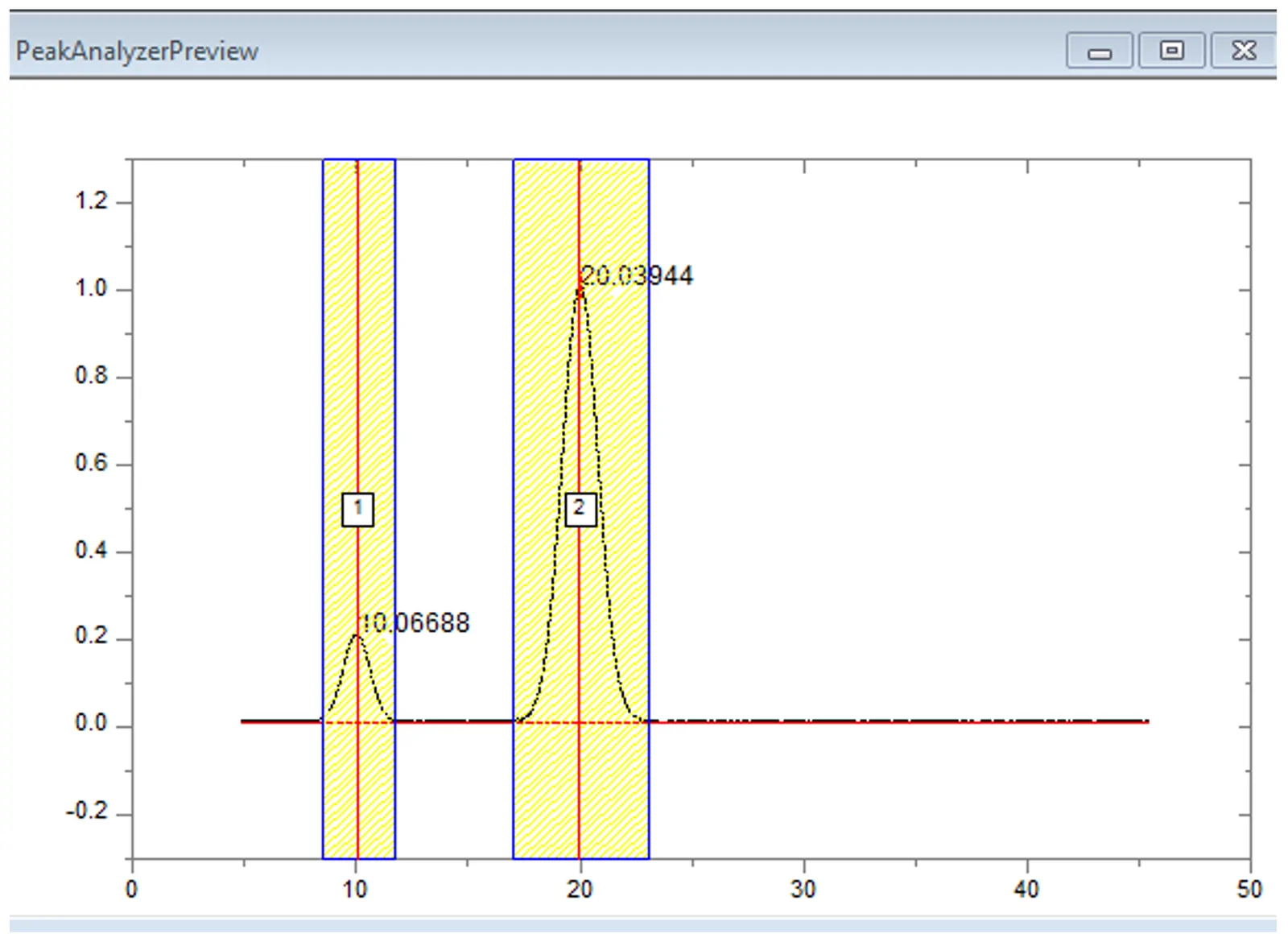
XRD crystallographic peaks for K2.

In all tested samples, X-ray crystallography revealed that two characteristic peaks for chitosan are observed: a peak at 2θ 9–10°, corresponding to partially ordered structures and appearing as a broad, significantly lower peak, and a peak at 2θ 19–20°, which is the main crystalline peak of chitosan. This second peak is also broad in most tested samples due to the semi-crystalline nature of chitosan, but it has a significantly higher intensity than the first peak (Figure 6). The peak positions are approximate, with minor shifts, and are located between 8.89 and 10.2° and between 19.7 and 20.1°, but the peak intensities and full widths at half maximum (FWHM) differ. The narrowest peaks were observed for samples K and K2: 1.42 at 10° and 1.89 at 20°, indicating the highest order within these samples. These are followed by samples UZ2 and 3DP, while the remaining samples are grouped together with FWHM values of 3–3.5 at 10° and 3.5–3.8 at 20°, which are large peak widths and indicate a high proportion of the amorphous phase in the chitosan structure. In all samples, the height and area of the peak at about 20° exceed those of the peak at 10°, with this difference being most pronounced in the most crystalline samples, K and K2. The appearance of the XRD crystallographic peaks, K as the most crystalline and 0DP2 as the most amorphous, is shown in Figure A1 and Figure A2 (Appendix A).

### 2.10. DPPH• Scavenging Ability

**Figure 7:**
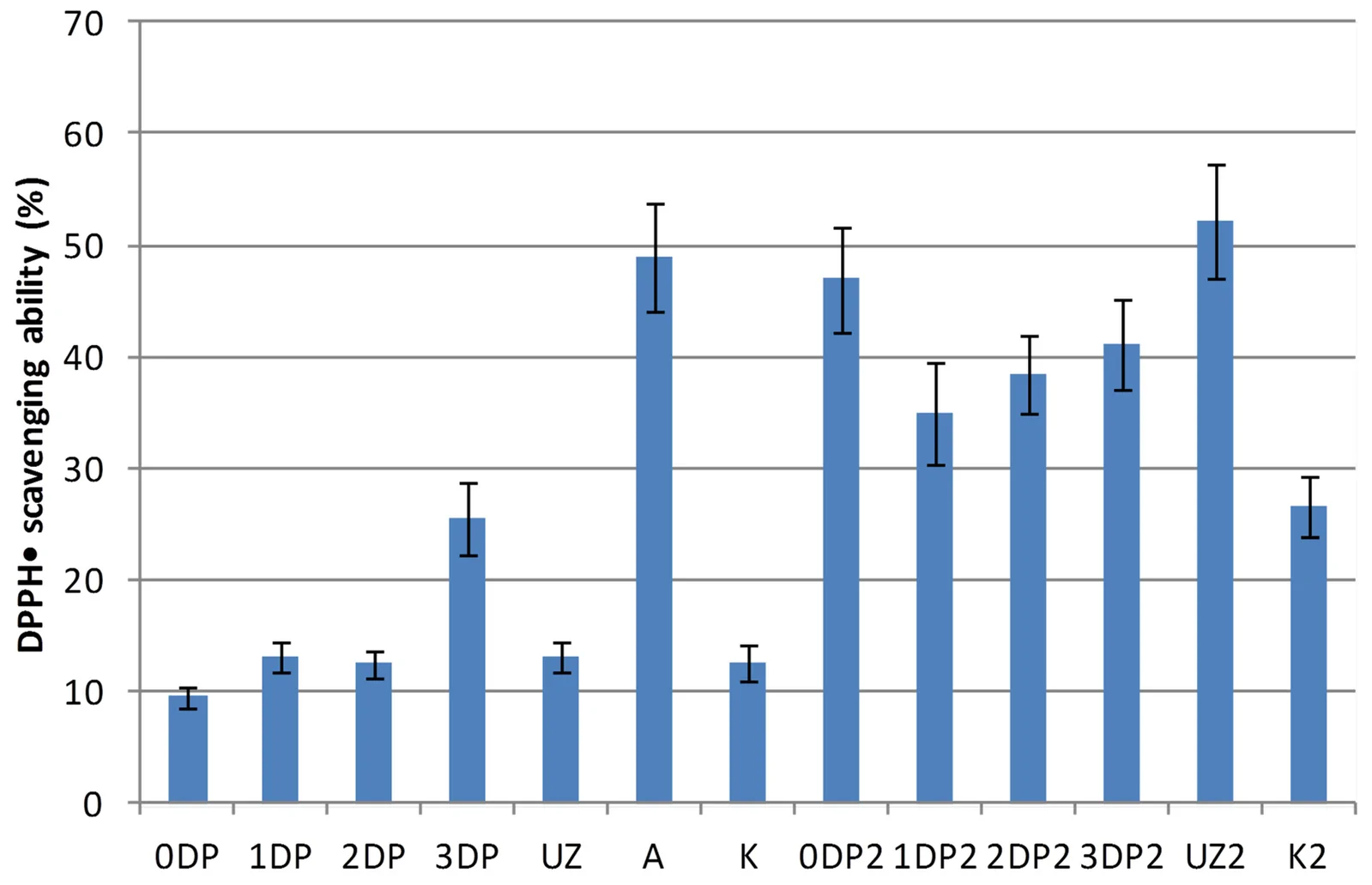
DPPH• scavenging ability of analyzed chitosan coating–forming matrices.

Within the DM1.3 series, DPPH• scavenging ability ranged from 9.49% to 12.58% for all coating–forming matrices (*p* > 0.05, no statistical difference), except for two coating–forming matrices, 3DP and A, where the antioxidant activity values were 25.43% and 48.84% (Figure 7). In the DM0.5 series, antioxidant activity values ranged from 34.95% to 46.93% (*p* < 0.05) for all coating–forming matrices except UZ2 and K2, for which the values were 52.16% and 26.62%. Possible reasons for the higher activity of the chitosan coating–forming matrix in the second series include more intense and thorough demineralization, resulting in a lower content of metals that can have a pro-oxidant effect, as well as greater availability of functional groups in chitosan from the DM0.5 series due to better solubility of the samples. Additionally, more intense acid treatment could lead to partial hydrolysis of the molecules and a greater proportion of shorter, more reactive chains. In samples 0DP2, UZ2 and A, according to FTIR analysis, it is reasonable to expect a contribution from the activity of residual proteins and/or peptides, as demonstrated by antioxidant activity. However, no statistically significant relationship was found between the total soluble protein content and DPPH radical scavenging activity for the DM1.3 series (Pearson’s r = 0.496, *p* = 0.257; Spearman’s ρ = 0.071, *p* = 0.906). Therefore, the antioxidant capacity is likely influenced by additional factors, such as the composition of the residual protein fraction, specific bioactive peptides, residual pigments, or chitosan structural characteristics. The DPPH• radical scavenging ability of chitosans depends on various factors, but numerous literature sources report a range of results, from no activity to about 25% DPPH scavenging ability with prolonged reaction time [18,19,20]. The higher values obtained for DM0.5 series samples, as well as the A sample from the DM1.3 series, might be attributed to residues of proteins, peptides, pigments and other low-molecular-weight active compounds, in combination with lower content of pro-oxidant metals and better solubility. The possible contribution of changes in chitosan molecular weight cannot be excluded.

### 2.11. Antimicrobial Activity

**Figure 8:**
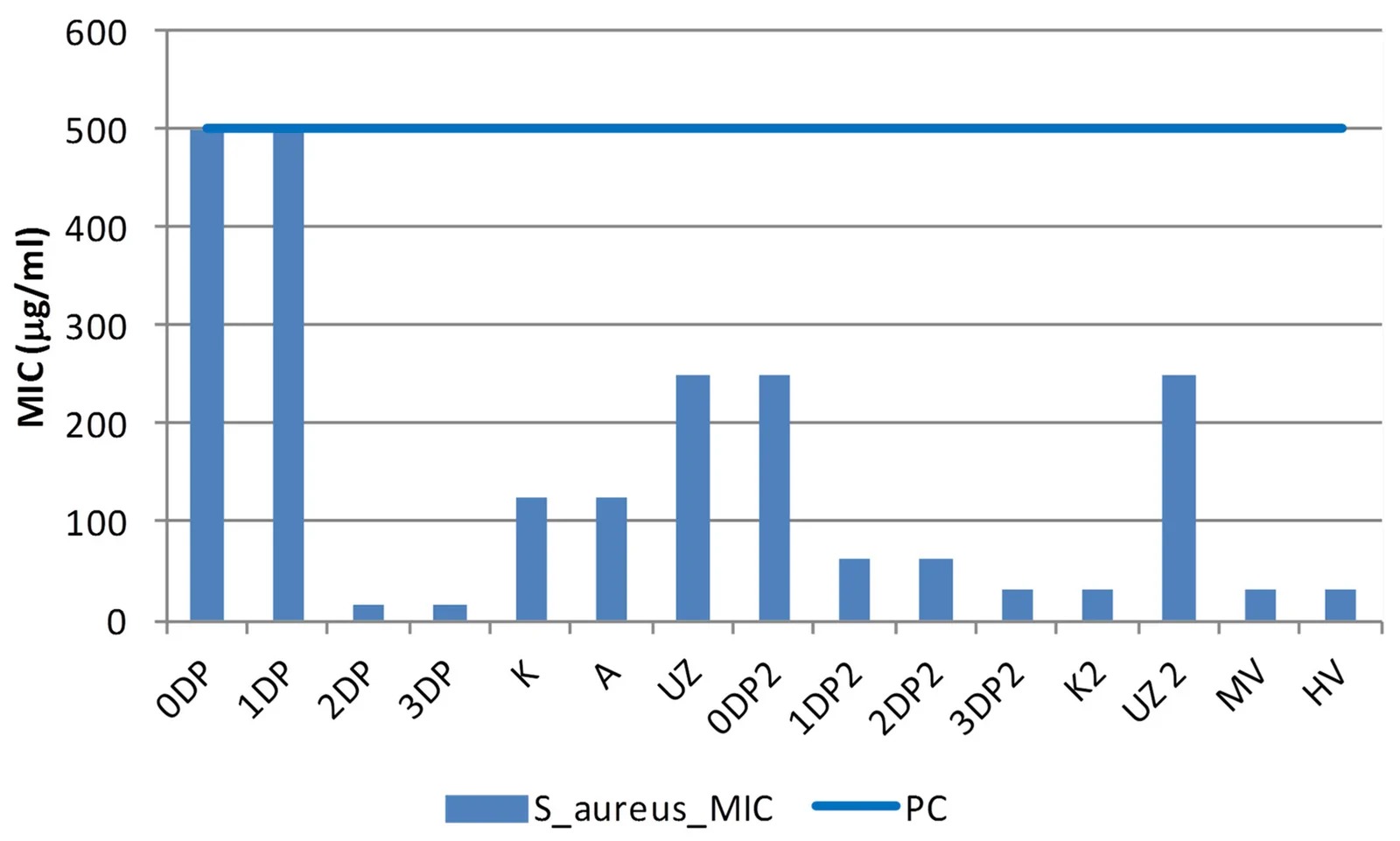
Minimal inhibitory concentration (MIC) (μg/mL) against *S. aureus* for the analyzed chitosan coating–forming matrices. MV and HV are commercial chitosan samples of medium and high viscosity. PC is the positive control, 1% acetic acid.

**Figure 9:**
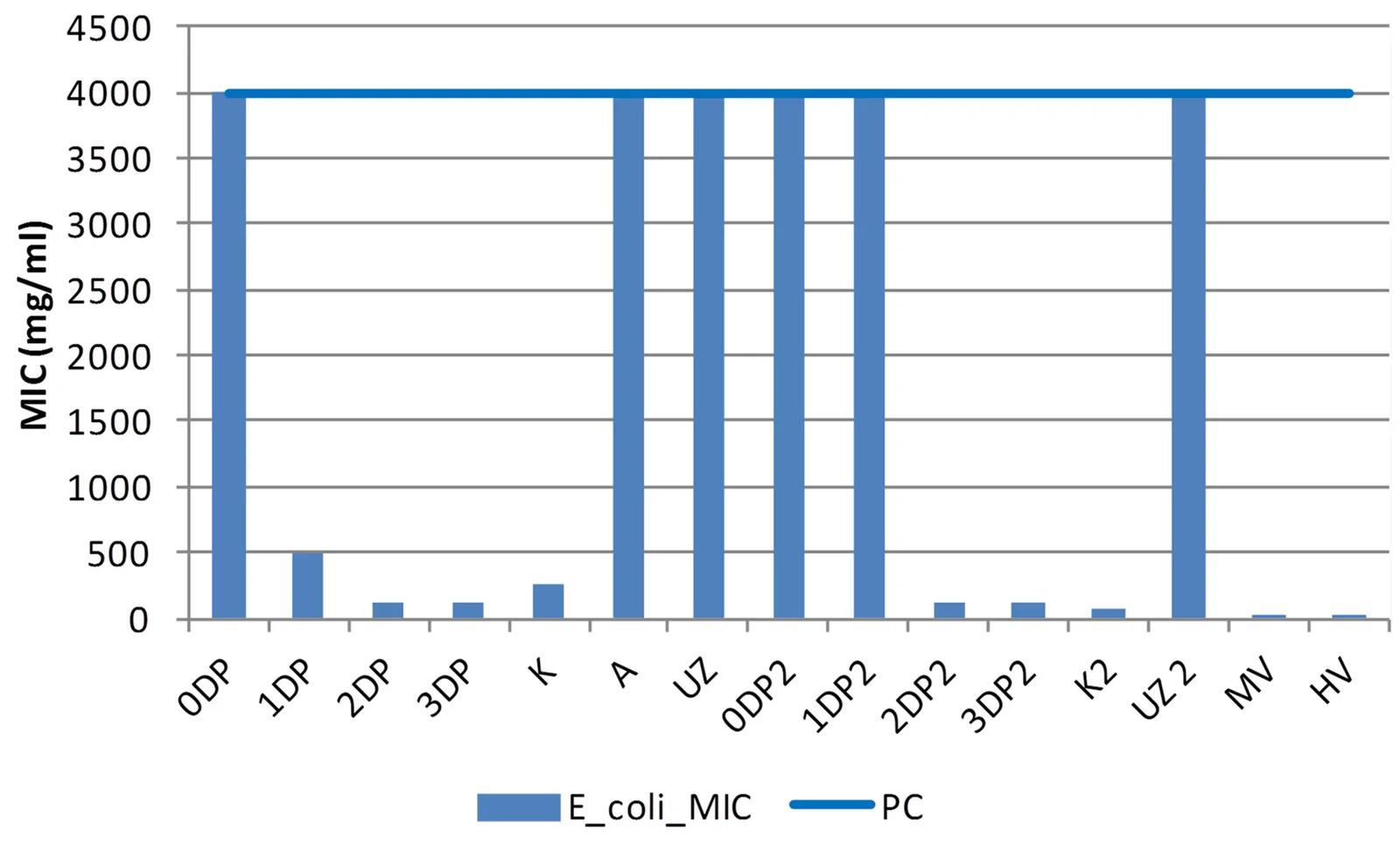
Minimal inhibitory concentration (MIC) (μg/mL) against *E. coli* for the analyzed chitosan coating–forming matrices. MV and HV are commercial chitosan samples of medium and high viscosity. PC is the positive control, 1% acetic acid.

The results for the antimicrobial activity of the coating–forming matrices of extracted chitosans are presented in Figure 8 and Figure 9. MV and HV are commercial medium- and high-viscosity chitosan samples. Considering the value of the positive control with *S. aureus* (1% acetic acid), except for samples 0DP and 1DP, the other samples exhibited varying levels of activity against this bacterium. It is possible that samples that are not completely pure, but contain residual proteins and other components attached to the charged, i.e., active groups of chitosan, show lower activity than the commercial samples. Molecular weight is also known to influence the antimicrobial activity of chitosan; its possible contribution cannot be excluded. Since molecular weight was not determined in the present study, no solid conclusions regarding its influence can be drawn. Regarding the activity of coating–forming matrices of isolated chitosans against *E. coli*, fewer samples demonstrated activity compared to Gram-positive *S. aureus*, which is consistent with the literature. Of the 13 experimental samples and two commercial samples, six did not show activity within the given concentration limits against *E. coli*. The remaining samples exhibited activity of varying intensity. The lowest MICs for *S. aureus* were observed in samples 3DP and 2DP (15.63 µg/mL and 20.84 µg/mL), followed by 3DP and K2 (31.25 µg/mL). For *E. coli*, the lowest MIC values were recorded for sample K2 (62.50 µg/mL), followed by 2DP, 3DP, 2DP2 and 3DP2 (125 µg/mL). Based on these results, samples 2DP and 3DP from both series, as well as sample K2, are identified as the most active in terms of antimicrobial activity.

MIC values for different chitosans reported in the literature vary significantly, as this value depends on several parameters: purity, molecular weight, degree of deacetylation, target microorganism, test conditions, and so on. Wang et al. (2021) reported an MIC of 30 μg/mL against Staphylococcus aureus and *Escherichia coli*, which in this study would be comparable to the commercial MV and HV samples and, to some extent, to the K2 sample [21]. In the work of Jung et al. (2010), the authors reported MIC values ranging from 0.03% to above 0.1% for acid-soluble chitosans, while for water-soluble chitosans, values ranged from 0.05% to above 0.8% for eight Gram-negative and six Gram-positive bacteria [22]. This range includes our 2DP and 3DP samples from both series, as well as the K2 sample. Ferreira et al. (2022) reported similar MIC values against *E. coli* (37.50 μg/mL), but even lower values for *S. aureus* (4.65 μg/mL) [23]. In the same paper, it is discussed that the range of reported MIC values, depending on the characteristics of chitosan, is extremely wide; values up to 1200 μg/mL and 1300 μg/mL are stated for these two bacteria, which would include all samples from this test except for six samples that had MIC values for *E. coli* greater than 4000 μg/mL.

### 2.12. Rheological Behavior and Wettability

The investigated chitosan solutions represent a viscoelastic chitosan polymer network, whose rheological behavior and wettability determine their ability to form continuous edible coatings. The results of rheological measurements of different chitosan coating–forming matrices are shown in Table 3. Within the group of samples DM1.3, increasing the number of deproteinization steps increases η_50_, accompanied by an increase in ST from 2.35 for sample 0DP to 5.11 for 3DP, a decrease in *n* from 0.5817 for 0DP to 0.3273 for 3DP, and an increase in *K* from 0.418 for 0DP to 2.046 for 3DP. This trend indicates that increasing the number of deproteinization steps results in more pronounced shear-thinning behavior and the formation of more structurally organized coating–forming matrices, which can be attributed to more efficient removal of proteins that interfere with the interchain interactions of chitosan, enabling a stronger connection and entanglement of the polymer chains [24,25]. The exception is sample 2DP, where this trend is not fully observed, which may indicate partial removal of protein components or the presence of structural inhomogeneities in the system.

Within the DM0.5 sample group, a different trend is observed. Increasing the number of deproteinization steps initially leads to an increase in the viscosity and structure of the viscoelastic chitosan polymer network for coating–forming matrices (1DP2). However, in samples with a higher number of steps (especially 3DP2), there is a decrease in the values of η_50_, ST, and *K*, accompanied by a simultaneous increase in the value of the exponent *n*. This behavior indicates a weakening of the shear-thinning character and a reduction in interchain interactions. These results suggest that the combination of aggressive demineralization (pH 0.5) and multistage deproteinization leads to partial degradation of chitosan chains, thereby reducing their ability to entangle.

By comparing samples from two different demineralization series with the same number of deproteinization steps, differences are observed. In samples 0DP, 1DP, and 2DP from both DM series, stronger demineralization resulted in a more viscous and structured viscoelastic chitosan polymer network for coating–forming matrices, probably due to higher solubility of chitosan and/or stronger interchain interactions. With samples 3DP and 3DP2, the situation is different. For these samples, η_50_, ST, and *K* decrease markedly, while *n* increases significantly. This indicates that strong demineralization followed by intense deproteinization most likely led to depolymerization and/or a substantial reduction in the entanglement of chitosan chains. Similar behavior was observed in samples UZ and UZ2.

According to the apparent viscosity values at a shear rate of 50 s^−1^, four subgroups can be identified. The highest values were measured for samples 3DP and 1DP2. However, a significant structural difference can be observed between these two samples. Sample 3DP exhibits a significantly higher ST, a lower exponent *n*, and a higher *K* value, indicating more pronounced shear-thinning behavior and greater structural organization of the viscoelastic chitosan polymer network. The second group comprises samples 0DP, 0DP2, 1DP, 2DP, and 2DP2. Within this group, differences in other parameters: ST, *K*, and *n*, also indicate variations in structural organization and shear-thinning behavior. The third subgroup consists of 3DP2 and UZ, while the group with the lowest apparent viscosity values at a shear rate of 50 s^−1^ includes samples UZ2, A, K, and K2. Among these samples with low η_50_, it is most likely that ultrasound and intensive chemical treatments caused depolymerization, while in sample A, the lower solubility of chitosan is probably due to residual proteins in the sample.

### 2.13. Contact Angle

The values of the contact angles for the analyzed viscoelastic chitosan polymer network for coating–forming matrices are given in Table 3. It can be observed that the contact angles for blueberries are the highest, which corresponds to the properties of the fruit surface, as blueberries have the most hydrophobic surface (wax). For contact angle values less than 90°, the liquid is considered to wet the surface, while values greater than 90° indicate that the liquid wets the examined surface poorly [26,27]. Considering the values obtained for the contact angles of the surfaces of different fruits with the tested chitosan coating–forming matrices, it can be seen that most of the analyzed matrices (except 0DP2, 1DP2 and 3DP) wet the nectarine and apple surfaces, which is suitable for the application of chitosan coatings. In the case of blueberries, the situation is different, and none of the analyzed solutions show good wetting ability on the surface of this fruit. Since the objective of this study was to compare the functional properties of chitosans obtained by different extraction processes using the same coating formulation, no further formulation optimization was performed. Nevertheless, these results indicate that modification of the coating–forming matrix composition, such as the incorporation of food-grade surfactants, adjustment of the formulation composition, or appropriate pretreatment of the fruit surface, would be required to improve wettability on blueberries and should be addressed in future studies.

Correlation analysis (Table 4) showed that the viscosity at 50 s^−1^ (η_50_) is the most significant parameter affecting the contact angle, especially for apple (r = 0.97 ***) and nectarine (r = 0.90 ***). The parameter *K* also shows a strong positive correlation, while ST and the exponent *n* have a moderate influence. For blueberry, the correlations are less pronounced, indicating the dominant influence of the fruit’s surface properties compared to the rheological characteristics of the coating–forming matrices.

### 2.14. Film-Forming Properties

**Figure 10:**
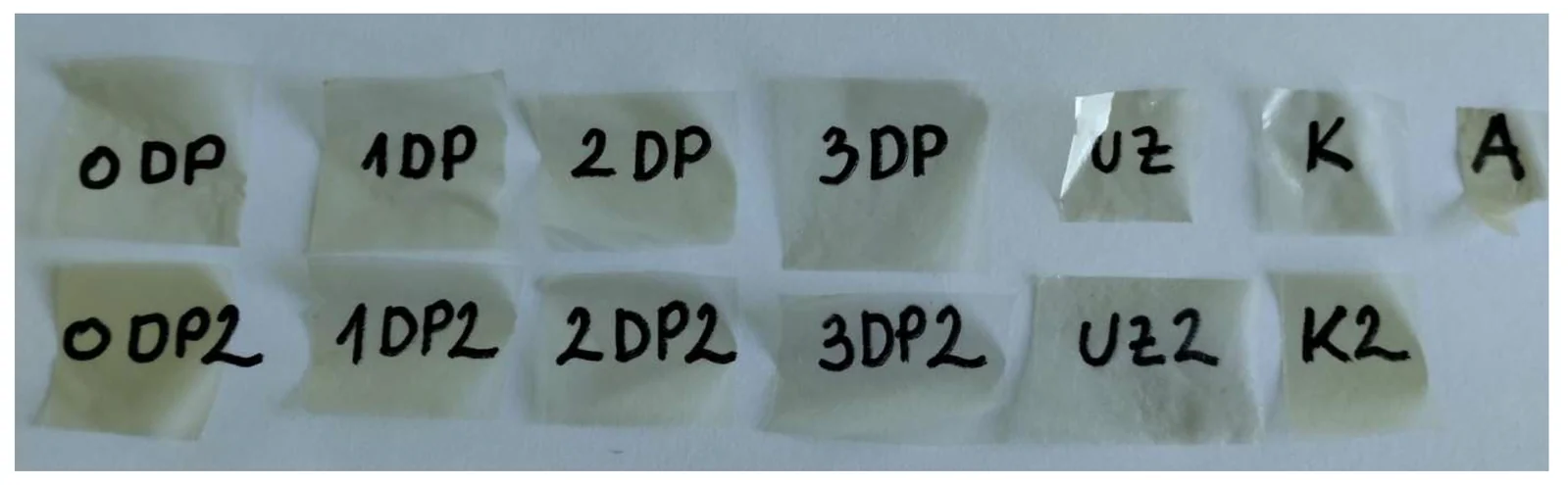
Appearance of films cast from different chitosan coating–forming matrices.

Chitosan films, 20–30 µm thick, were obtained by casting from all chitosan coating–forming matrices. This confirms the film-forming properties of all formulations. The films are transparent and slightly opalescent, with a whitish to yellowish tint (Figure 10). It was observed that films A and UZ 2, cast from matrices with the lowest values of the rheological parameters η_50_ and K, are the thinnest and weakest, rupturing during handling. This may be due to the poorer solubility of these chitosans, leading to the formation of a less coherent and less connected structure. Among the tested samples, the film obtained from the 3DP solution stands out, characterized by the highest values of η_50_, ST and K, as well as the lowest value of the exponent n. This film exhibits increased strength and compactness, manifested by greater thickness and a more pronounced gloss, but also increased brittleness, observed during cutting and handling. The rheological parameters of the chitosan coating–forming matrices indicate the degree of interchain interactions in the matrices, which is reflected in the mechanical properties of the resulting films. This observation highlights the close relationship between the viscoelastic behavior of hydrated chitosan matrices and the performance of the resulting edible coating films. Other chitosan coating–forming matrices, which did not show these extreme values of rheological parameters, produced more elastic films that could be handled without breaking [25,28]. The present study focused on evaluating the intrinsic film-forming capability of chitosans obtained by different extraction processes. Continuous transparent films were successfully obtained from most samples, demonstrating their ability to form coherent polymeric matrices. However, comprehensive characterization of the resulting films, including mechanical and barrier properties, was beyond the scope of this work and will be addressed in future studies aimed at developing optimized edible coating formulations.

## 3. Conclusions

The results show that the demineralization conditions and the number of deproteinization steps significantly influence the properties of chitosan isolated from *F. limosus* crayfish shell. More intense demineralization (pH 0.5) enabled more efficient removal of minerals, especially calcium, but, when combined with multi-stage deproteinization, led to a reduction in interchain interactions in the viscoelastic chitosan polymer network for coating–forming matrices. Increasing the number of deproteinization steps decreased the protein content of the samples, as confirmed by amino acid analysis and FTIR spectra. All samples had a similar degree of deacetylation (70–75%), while differences in crystallinity, rheological properties, and antioxidant and antimicrobial activity were related to the processing methods. Samples subjected to more intensive deproteinization produced coating–forming matrices that showed more pronounced antimicrobial activity, while coating–forming matrices from the more intensively demineralized series, DM0.5, as well as sample A, had higher antioxidant activity, probably due to the presence of residual bioactive components and lower levels of pro-oxidative metals. All tested formulations were able to form films and showed potential for use as edible coatings, with most formulations demonstrating good wetting properties on apple and nectarine surfaces. The results indicate that optimizing demineralization and deproteinization conditions allows the production of chitosan with different functional properties suitable for application in food and postharvest technology.

## 4. Materials and Methods

### 4.1. Materials

Commercial chitosan of high viscosity, medium viscosity and low viscosity (hv, mv and lv) was purchased from Sigma-Aldrich Chemical Co. (St. Louis, MO, USA). For FTIR and color analysis, chitosans 70/1000, 80/1000 and 90/1000 were used as reference material (Heppe Medical Chitosan GmbH, Halle (Saale), Germany). The following reagents and raw materials were used to carry out the chitosan extraction experiment: ground crayfish shell (*Faxonius limosus*), granulation < 0.5 mm; granulated NaOH; deionized water; HCl; acetic acid. All used chemicals were of analytical grade.

Crayfish were sourced from the Danube River. Their shells were obtained after the edible contents were extracted at the Institute of Food Technology in Novi Sad. The crayfish were boiled in water for 10 min, after which the meat was removed and the shells were dried in a drying oven at 55–65 °C. The shells were then sterilized using a UV lamp for 12 h. Subsequently, the shells were ground, sieved through appropriate sieves, and prepared for further steps in the process of obtaining chitosan.

### 4.2. Chitosan Extraction Processes

During the demineralization phase, dried and ground crayfish shells were treated with 0.5 M HCl at room temperature at a ratio of 1:12 (shell to acid) on a magnetic stirrer for 45 min, while monitoring pH changes. One series of samples (DM1.3) was demineralized until the pH reached 1.3, requiring one acid bath. To further lower the pH, samples were filtered, washed with distilled water, and the acid bath was repeated. This produced the sample series DM0.5, with the pH in the demineralization step reaching 0.5. The pH values of 1.3 and 0.5 were not selected a priori but resulted from the progression of the demineralization process. After the first HCl treatment, vigorous gas evolution was observed, indicating intensive dissolution of calcium carbonate, and the pH stabilized at approximately 1.3. To further intensify demineralization, the solid residue was subjected to a second acid treatment. During this stage, gas evolution was minimal, indicating that most mineral components had already been removed, and the pH of the suspension stabilized at approximately 0.5. These two demineralization stages were therefore used to investigate the influence of demineralization intensity on the subsequent properties of the isolated chitosans. After demineralization, the samples were filtered and the precipitate was washed with deionized water, with repeated filtration. The washed precipitate was dried in a drying oven at 55–65 °C for 2 h.

The deproteinization phase involved alkaline treatment with 0.3 M NaOH at a ratio of 1:10 (shell to base), at 80–85 °C with constant stirring on a magnetic stirrer. This phase was repeated 0 to 3 times, yielding the samples 0DP, 1DP, 2DP, and 3DP. After completion, rinsing with distilled water and filtration were performed, and the washed precipitate was dried in an oven at 55–65 °C for 2 h. Two alternative deproteinization methods were also applied. One method (A) used autolysis for protein degradation [29]. Autolysis was promoted by exposing a 1:1 (*w*/*v*) shell powder–water homogenate to UV light (253.7 nm, 20 W) for 20 min. The treated homogenate was then adjusted to obtain a 20% (*w*/*v*) suspension at pH 9 and incubated under a stepwise temperature regime, beginning at 40 °C and increasing by 5 °C every hour until 65 °C was reached. After 7 h, enzymatic activity was stopped by heating the suspension to 100 °C for 10 min, after which it was allowed to cool to room temperature.

The other alternative deproteinization method involved ultrasound treatment with a significantly reduced amount of NaOH, producing the UZ samples.

Deacetylation was carried out by adding 50% NaOH at a chitin:NaOH ratio of 1:20, followed by thermal treatment at 125 °C in an oil bath with glycerol for 1.5 h. The sample was then filtered and washed with distilled water. The washed precipitate was dried in an oven at 55–65 °C for 2 h. In control (K) samples, pigments were removed from isolated 3DP chitosans using a 15:1 *v*/*w* ratio of 70% ethanol to chitosan in a bath for 60 min at 40 °C, followed by washing with water and drying. The chitosan was then depigmented with 3% H_2_O_2_ for 3 h at 50 °C in a 20:1 *v*/*w* solvent/chitosan ratio, washed with water, filtered, and dried. A flowchart of the process is given. Samples were labeled as shown in Table 5.

### 4.3. The Yield of Chitosan from Different Extraction Processes

The yield of chitosan from different extraction processes was calculated using the equation:

(1) Chitosan Yield (%) = m_chitosan/m_shell ×100 Pre deacetylation material Yield (%) = m_ pre_deacetylation_ material /m_shell ×100 Chitosan recovery from pre-deacetylation material (%) = m_chitosan/m_ pre_deacetylation_ material ×100

For each sample, the test was repeated three times, and the result was presented as mean ± SD.

The yield of the intermediate material before deacetylation was calculated relative to the initial shell mass. For 0DP and 0DP2, this material represented the demineralized shell obtained at pH 1.3 and pH 0.5, respectively, because no deproteinization step was applied. For the remaining samples, it represented the material obtained after demineralization followed by the corresponding deproteinization treatment: one, two or three conventional alkaline steps, ultrasound-assisted deproteinization, autolysis-assisted deproteinization, or the additional treatments used for K and K2.

### 4.4. The Chitosans Moisture Content

The moisture content was determined gravimetrically by drying the samples in an oven at 70 °C to constant mass:

(2) MC(%) = 100·((m2−(m3−m1))/m2)

where (m1) is the mass of the dry empty container, (m2) is the mass of the sample before drying in the oven and (m3) is the total mass after drying to constant mass. For each sample, the test was repeated three times, and the result was presented as mean ± SD.

### 4.5. The Degree of Deacetylation

The degree of deacetylation of extracted chitosan samples was determined as described by Yuan et al. (2011) using potentiometric titration [30]. Chitosan samples (0.5 g) were dissolved in 20 mL of 0.3 N HCl, and 400 mL of distilled water was added after dissolution. Titration was carried out with a 1 N NaOH solution. A titration curve of pH versus NaOH titration volume was generated, and the curve’s inflection points were identified. The volume of NaOH at each inflection point was applied to the following equation:

(3) NH_2_% = 16.1 · (y − x)/m

where m is the weight of chitosan used, x is the first inflection point, and y is the second inflection point. For each sample, the test was repeated three times, and the results were presented as mean ± SD.

### 4.6. Determination of Total Amino Acids (TAA)

The total amino acid composition of the chitosan powder samples was determined by ion-exchange chromatography using an Automatic Amino Acid Analyzer Biochrom 30+ (Biochrom, Cambridge, UK), following the procedure described in the literature [31]. Separation was performed on a strong cation-exchange column using a sodium-based buffer system consisting of four eluents with pH values ranging from 3.2 to 9.2, together with a sodium hydroxide regeneration solution, at a flow rate of 35 mL h^−1^. Amino acids were detected after post-column derivatization with ninhydrin, with absorbance monitored at 570 nm, except for proline, which was measured at 440 nm.

Prior to chromatographic analysis, the samples were hydrolyzed in 6 M HCl (Merck KGaA, Darmstadt, Germany) at 110 °C for 24 h. The hydrolysates were cooled to room temperature, dissolved in 25 mL of loading buffer (pH 2.2; Biochrom, Cambridge, UK), and filtered through a 0.22 μm PTFE membrane filter (Membrane Solutions LLC, Plano, TX, USA). The filtrates were transferred into Agilent autosampler vials and stored under refrigerated conditions until analysis. Amino acids were identified by comparing their retention times with those of a certified amino acid standard mixture (Amino Acid Standard Solution, Sigma-Aldrich, St. Louis, MO, USA). The results were expressed as grams of amino acid per 100 g of sample.

For each sample, the test was run in duplicate, and the result was presented as mean ± SD.

### 4.7. Lowry Method for Soluble Protein Content

Soluble protein content in the extracted chitosan samples was measured using the Lowry method [32]. Samples were suspended in distilled water at a ratio of 1:10, and 50% NaOH was added to adjust the pH to 12 and maintain it for a few minutes. The samples were then incubated at 80 °C for 20 min, filtered, and the filtrate was used to analyze soluble proteins. For each sample, the test was repeated three times, and the results were presented as mean ± SD.

### 4.8. Color Parameters

The color of the chitosan powder samples was evaluated using a CR-400 Chroma Meter (Konica Minolta Inc., Osaka, Japan) equipped with an 8 mm measuring aperture and calibrated with the standard white calibration plate (CR-A33b). Measurements were performed under D65 illumination using a 2° standard observer angle. Instrument calibration was carried out before each series of measurements. Color was expressed in the CIELAB color space, where L* denotes lightness, a* the red–green coordinate, and b* the yellow–blue coordinate [33]. In addition to the primary CIELAB parameters, chroma (C)* and hue angle (h) were calculated. Chroma (C*) describes the saturation or intensity of color and was calculated according to the following equation:

(4) C* = √((a*)^2^ + (b*)^2^),

while the hue angle is calculated as follows:

(5) h° = tan^(−1)^ (b*/a*)

Higher C* values indicate greater color saturation, while the hue angle defines the dominant color tone in the CIELAB space.

### 4.9. Metal Content Determination

The contents of Pb and Cd were determined by atomic absorption spectrometry (AAS) using a flame atomic absorption spectrometer (Varian SPECTRA AA-10, Varian Techtron Pty Limited, Mulgrave, Victoria, Australia) equipped with a flame furnace and operated with an air-acetylene flame after dry ashing according to [34] with minor modifications. The rest of the metals, Zn, Cu, Fe, Mg, K, Na, Ca, Mn and Hg were determined by atomic absorption spectrometry (AAS) using method [35]. For each sample, the test was run in duplicate, and the result was presented as mean ± SD. For each sample, the test was repeated three times, and the results were presented as mean ± SD.

### 4.10. FT-IR Spectrophotometry

FTIR spectroscopy was employed to investigate the structural properties of the chitosan powders. Measurements were carried out using a Nicolet iS10 FT-IR spectrometer (Thermo Fisher Scientific, Waltham, MA, USA) fitted with an ATR accessory. Spectra were collected in the 4000–400 cm^−1^ region with a spectral resolution of 4 cm^−1^, averaging 16 scans for each measurement. Data acquisition and spectral processing were performed using OMNIC 8.1 software (Thermo Fisher Scientific, Waltham, MA, USA).

### 4.11. X-Ray Diffraction

X-ray diffraction (Rigaku MiniFlex 600diffractometer, Tokyo, Japan) analysis of extracted chitosan powder samples was performed using Cu-Kα radiation in the range from 10 to 80° with a step of 0.02° and a dwell time of 3 s.

### 4.12. 2,2-Diphenyl-1-Picrylhydrazyl (DPPH) Assay of the Chitosan Coating–Forming Matrices

The antioxidant activity of chitosan samples was determined using the DPPH radical scavenging assay described by Šuput et al. [36]. Chitosan coating–forming matrices 1% *w*/*v* in 1% acetic acid were incubated in the dark for 24 h at room temperature in a freshly prepared DPPH• solution (0.0185 g DPPH• dissolved in 50 mL ethanol and subsequently diluted with ethanol at a ratio of 1:5), and stirred during this period. After incubation, the remaining DPPH• was quantified by measuring the absorbance at 520 nm using a T80/T80+ UV–Vis spectrophotometer (PG Instruments Ltd., Lutterworth, UK). Radical scavenging activity was expressed as antioxidant activity (AO, %) and calculated according to Equation (6):

(6) AO (%) = (DPPH•_0_ − DPPH•s)/(DPPH•_0_) × 100

where DPPH•_s_ represents the DPPH• concentration in the sample containing chitosan, whereas DPPH•_0_ corresponds to the blank solution prepared without chitosan, only 1% acetic acid. Each sample was analyzed in triplicate, and the results are presented as mean ± standard deviation.

### 4.13. The Antimicrobial Activity of the Chitosan Coating–Forming Matrices

The antimicrobial activity of the chitosan coating–forming matrix samples was evaluated against the Gram-positive bacterium *Staphylococcus aureus* ATCC 25923 (American Type Culture Collection, Rockville, MD, USA) and the Gram-negative bacterium *Escherichia coli* ATCC 10536 (American Type Culture Collection, Rockville, MD, USA). Both reference strains were obtained from the Culture Collection of the Microbiological Laboratory, Institute of Food Technology, University of Novi Sad (Serbia). Stock cultures were maintained at −80 °C in Trypto-Casein Soy Broth (TSB, Biokar, Beauvais, France) containing 40% glycerol. Prior to antimicrobial testing, the strains were recovered by streaking onto Tryptone Soya Agar (TSA, Oxoid CM0131, Hampshire, UK) and incubating the plates at 37 °C for 24 h.

A bacterial suspension adjusted to 1 × 10^6^ CFU/mL was prepared, and an aliquot of 100 μL was dispensed into each well of the microtiter plate with Mueller–Hinton Broth (MHB, HiMedia, Mumbai, India) and chitosan concentrations ranging from 4000 to 7.81. Following inoculation, the plates were incubated at 37 °C for 24 h. Each experiment included a positive control (culture medium inoculated with the reference strain without chitosan, only 1% acetic acid) and a negative control (sterile culture medium without bacterial inoculation). All measurements were performed in four independent replicates.

After incubation, 20 μL of 0.01% resazurin solution (7-hydroxy-3H-phenoxazin-3-one 10-oxide; HiMedia Laboratories Pvt. Ltd., Thane, Maharashtra, India) was added to each well as a bacterial growth indicator, and the plates were incubated for an additional 24 h. The minimum inhibitory concentration (MIC) was defined as the lowest concentration of chitosan that prevented visible bacterial growth, corresponding to the first well showing no color change [37].

Assays were carried out in four independent replicates for each tested microorganism.

### 4.14. Rheological Measurements

Rheological measurements were performed using a RheoStress 600HP rheometer (Thermo Electron (Karlsruhe) GmbH, Karlsruhe, Germany), at 20 °C. The rheological behavior of the coating–forming matrix samples was evaluated using a rotational rheometer equipped with a cone–plate measuring geometry (gap: 0.052 mm; sample volume: 1 mL). Measurements were carried out at 20 °C under controlled shear rate (CR) conditions. Prior to analysis, the samples were equilibrated at the measuring temperature for 500 s. The shear protocol consisted of three consecutive steps: (i) an upward shear rate ramp from 0 to 1000 s^−1^ over 120 s, (ii) maintaining the maximum shear rate for 100 s, and (iii) a downward shear rate ramp from 1000 to 0 s^−1^ over 120 s. The flow behavior and viscosity changes were monitored throughout the measurement.

The flow behavior of chitosan coating–forming matrices was described using the Ostwald–de Waele (power-law) model:

(7) $$\tau =K\cdot {\dot{\gamma }}^{n}$$

where τ is the shear stress (Pa), $\dot{\gamma }$ is the shear rate (s^−1^), *K* is the consistency index (Pa·s^n^), and *n* is the flow behavior index.

The apparent viscosity at a shear rate of 50 s^−1^ (η_50_) was used as a reference parameter for comparison among samples, as it represents the viscosity under moderate shear conditions relevant for coating applications.

The shear-thinning index (ST) was calculated as the ratio of apparent viscosities at low and high shear rates:

(8) ST = η_50_/η_500_

where η_50_ and η_500_ are the apparent viscosities at shear rates of 50 s^−1^ and 500 s^−1^, respectively. This parameter was used to quantify the degree of shear-thinning behavior.

The flow behavior index (*n*) was obtained from the slope of the log–log plot of shear stress versus shear rate.

Correlation analysis was performed to evaluate the relationship between rheological parameters (η_50_, ST, *n*, and *K*) and contact angle values measured on fruit surfaces. Pearson correlation coefficients were calculated using Microsoft Excel. The statistical significance of the correlation coefficients was evaluated using a two-tailed t-test, and correlations were considered statistically significant at *p* < 0.05.

### 4.15. Contact Angle Measurement

Contact angles on the surfaces of three different fruits—apple, nectarine, and blueberry—were measured using the contact angle measurement in ImageJ software 1.54 g with the DropSnake plugin, following the method described and validated by Senturk Parreidt et al. (2017) [38]. At least three independent measurements were performed, and the result was given as the average with the indicated standard deviation.

### 4.16. Chitosan Film Formation Procedure and Film Thickness Measurement

The casting method was used to form films from chitosan film-forming solutions, which were obtained in the same way as appropriate coating–forming matrices. The solution was cast onto a Petri dish coated with Teflon cloth using a mass per surface ratio of 0.32 g/cm^2^ and left to dry at room temperature. Dry films were peeled and stored in a refrigerator.

Film thickness was measured using a micrometer Digico 1, with a sensitivity of 0.001 mm (Tesa, Renens, Switzerland). Thickness was measured at 5 points for each film.

### 4.17. Statistical Analysis

Statistical analysis was conducted using OriginPro 8 (OriginLab Corporation, Northampton, MA, USA). Data were presented as mean values with their standard deviation indicated (mean ± SD). Variance analysis (ANOVA) was performed with a confidence interval of 95% (*p* < 0.05). Means were compared using the Tukey test.

Pearson’s and Spearman’s correlation analyses were performed to assess the association between soluble protein content and DPPH radical scavenging activity using the mean values of the seven samples. Differences were considered statistically significant at *p* < 0.05. In addition, the effects of the demineralization series (DM1.3 and DM0.5), extraction treatment (0DP, 1DP, 2DP, 3DP, K and UZ), and their interaction on chitosan yield, moisture content and degree of deacetylation were evaluated using two-way analysis of variance (ANOVA). When significant differences were detected, Tukey’s honestly significant difference (HSD) test was applied for multiple comparisons at a significance level of *p* < 0.05. Analyses were performed in jamovi, version 2.7.30.

## Figures and Tables

**Table 1:** Yields of pre-deacetylation material and final chitosan obtained by different extraction processes.

Sample	Demineralization Endpoint	Deproteinization Treatment	Pre-Deacetylation Material Yield (%)	Final Chitosan Yield (%)	Chitosan Recovery from Pre-Deacetylation Material (%)	The Moisture Content (%)	The Degree of Deacetylation (%)
0DP	pH 1.3	No deproteinization	43.00 ± 6.45	10.15 ± 1.03	23.60	4.56 ± 0.08 ^a^	69.9 ± 6.92
1DP	pH 1.3	One conventional alkaline deproteinization step	26.78 ± 3.21	12.45 ± 1.25	46.49	4.85 ± 0.53 ^a^	71.9 ± 8.13
2DP	pH 1.3	Two conventional alkaline deproteinization steps	21.08 ± 2.74	10.68 ± 1.09	50.66	4.83 ± 0.16 ^a^	72.2 ± 11.23
3DP	pH 1.3	Three conventional alkaline deproteinization steps	14.74 ± 1.62	9.42 ± 0.95	63.91	3.87 ± 0.16 ^ab^	70.3 ± 6.96
K	pH 1.3	Three conventional alkaline deproteinization steps, followed by defatting and depigmentation	11.50 ± 1.38	10.47 ± 1.03	91.04	3.46 ± 0.38 ^b^	69.9 ± 8.90
UZ	pH 1.3	Ultrasound-assisted deproteinization	27.85 ± 4.18	13.81 ± 1.33	49.59	3.86 ± 0.07 ^ab^	71.2 ± 12.08
A	pH 1.3	Autolysis-assisted deproteinization	26.11 ± 3.92	13.82 ± 1.40	52.93	3.51 ± 0.22 ^b^	73.6 ± 5.15
0DP2	pH 0.5	No deproteinization	37.00 ± 6.29	9.41 ± 0.96	25.44	5.01 ± 0.41 ^a^	68.9 ± 8.77
1DP2	pH 0.5	One conventional alkaline deproteinization step	21.52 ± 3.01	7.64 ± 0.80	35.48	6.13 ± 0.22 ^b^	71.0 ± 10.04
2DP2	pH 0.5	Two conventional alkaline deproteinization steps	16.36 ± 2.45	7.12 ± 0.75	43.52	7.04 ± 0.16 ^bc^	71.6 ± 8.10
3DP2	pH 0.5	Three conventional alkaline deproteinization steps	15.00 ± 1.43	6.27 ± 0.69	41.80	7.49 ± 0.28 ^c^	72.9 ± 12.37
K2	pH 0.5	Three conventional alkaline deproteinization steps, followed by defatting and depigmentation	7.24 ± 0.87	6.57 ± 0.69	90.81	6.40 ± 0.07 ^b^	73.7 ± 8.34
UZ2	pH 0.5	Ultrasound-assisted deproteinization	22.89 ± 3.43	8.19 ± 0.79	35.80	4.96 ± 0.01 ^a^	68.9 ± 10.72

Different lowercase letters within the same demineralization series indicate significant differences according to Tukey’s post hoc test (*p* < 0.05).

**Table 2:** Total amino acids (TAA) composition of the representative samples from the DM 1.3 series obtained without deproteinization (0DP), after one-, two- and three-step deproteinization (1DP, 2DP and 3DP, respectively), and after autolysis-assisted deproteinization (A), expressed as g/100 g of sample.

Amino Acids	0DP	1DP	2DP	3DP	A
Aspartic acid	0.0316 ± 0.01	0.0208 ± 0.00	0.0432 ± 0.01	n.d.	0.0267 ± 0.00
Threonine	n.d.	n.d.	n.d.	n.d.	n.d.
Serine	0.0072 ± 0.00	n.d.	n.d.	n.d.	n.d.
Glutamic acid	0.0060 ± 0.00	n.d.	n.d.	n.d.	0.0110 ± 0.00
Proline	n.d.	n.d.	n.d.	n.d.	n.d.
Glycine	0.0039 ± 0.00	0.0048 ± 0.00	0.0013 ± 0.00	0.0013 ± 0.00	0.0061 ± 0.00
Alanine	n.d.	n.d.	0.0233 ± 0.00	n.d.	0.0003 ± 0.00
Cystine	0.0455 ± 0.01	0.0404 ± 0.01	n.d.	0.0241 ± 0.00	0.0342 ± 0.01
Valine	0.0370 ± 0.01	0.0263 ± 0.00	n.d.	n.d.	0.0134 ± 0.00
Methionine	n.d.	n.d.	n.d.	n.d.	0.0077 ± 0.00
Isoleucine	n.d.	n.d.	n.d.	n.d.	0.0181 ± 0.00
Leucine	n.d.	n.d.	n.d.	n.d.	0.0163 ± 0.00
Tyrosine	n.d.	n.d.	n.d.	n.d.	n.d.
Phenylalanine	n.d.	n.d.	n.d.	n.d.	0.0233 ± 0.00
GABA	0.0012 ± 0.00	0.0016 ± 0.00	0.0013 ± 0.00	0.0055 ± 0.00	0.0037 ± 0.00
Histidine	0.1674 ± 0.02	0.2030 ± 0.02	0.0958 ± 0.02	0.1090 ± 0.02	0.0651 ± 0.01
Lysine	0.0196 ± 0.00	n.d.	n.d.	n.d.	0.0028 ± 0.00
Arginine	n.d.	n.d.	n.d.	n.d.	n.d.
Taurine	0.0414 ± 0.01	0.0493 ± 0.01	0.0492 ± 0.01	0.0403 ± 0.01	0.0422 ± 0.01

n.d.—not detected.

**Table 3:** Viscoelastic chitosan polymer network for coating–forming matrices rheological parameters: apparent viscosity η at shear rates 50 s^−1^, 100 s^−1^ and 500 s^−1^; shear-thinning index ST; flow behavior index *n*, consistency index *K* (Pa·s^n^) and contact angle values θ (°) for.

	η (Pa·s) at $\dot{\gamma }$ = 500, 100 and 50 s^−1^			ST	*n*	*K* (Pa·s^n^)	Nectarine θ in °	Apple θ in °	Blueberry θ in °
$\dot{\gamma }$ in 1/s	500	100	50						
0DP	0.03125	0.05447	0.07239	2.32	0.5817	0.418	74.44 ± 1.87	77.27 ± 2.18	107.50 ± 1.18
0DP2	0.02946	0.06534	0.09890	3.36	0.5134	0.611	98.80 ± 0.14	87.36 ± 0.69	104.99 ± 1.45
1DP	0.01775	0.04461	0.07240	4.08	0.4094	0.736	85.60 ± 2.83	83.91 ± 2.66	104.34 ± 5.06
1DP2	0.05599	0.10880	0.15244	2.72	0.5887	0.727	108.10 ± 5.94	107.50 ± 1.18	111.96 ± 6.65
2DP	0.04014	0.07096	0.09488	2.36	0.5575	0.624	79.04 ± 0.11	80.06 ± 7.24	102.08 ± 4.68
2DP2	0.04502	0.08121	0.10420	2.41	0.5406	0.650	78.96 ± 1.89	84.08 ± 5.79	104.06 ± 5.21
3DP	0.02995	0.09755	0.15320	5.11	0.3273	2.046	109.55 ± 1.20	98.80 ± 0.10	117.74 ± 5.45
3DP2	0.02143	0.03266	0.04030	1.88	0.7307	0.115	74.80 ± 1.56	69.53 ± 2.47	111.96 ± 6.65
UZ	0.01665	0.02738	0.03578	2.15	0.6794	0.123	74.88 ± 0.26	69.18 ± 2.28	107.52 ± 1.90
UZ2	0.00399	0.00628	0.00835	2.09	0.7074	0.025	66.13 ± 4.48	66.54 ± 2.04	98.23 ± 24.41
A	0.00414	0.00622	0.00800	1.93	0.7405	0.021	56.85 ± 4.03	64.41 ± 2.15	98.24 ± 8.52
K	0.00461	0.00773	0.01050	2.28	0.6710	0.036	74.44 ± 1.87	67.64 ± 1.74	103.86 ± 2.09
K2	0.00901	0.01319	0.01628	1.81	0.7560	0.041	68.59 ± 1.17	66.18 ± 2.06	103.56 ± 2.86

**Table 4:** Correlation analysis between rheological parameters (η_50_, ST, *n*, and *K*) and contact angle on fruit surfaces.

	Correlation		
	Nectarine	Apple	Blueberry
η_50_	0.90181 ***	0.96606 ***	0.66045 *
ST	0.76155 **	0.68344 *	0.66045 *
*n*	−0.76610 **	−0.73417 **	−0.49753
*K*	0.80588 ***	0.79514 **	0.66851 *

Values represent Pearson correlation coefficients (r). * *p* < 0.05; ** *p* < 0.01; *** *p* < 0.001.

**Table 5:** Chitosan sample labeling.

Sample	Demineralization	Deproteinization
0DP	to pH 1.3	no
1DP	to pH 1.3	1 base bath
2DP	to pH 1.3	2 base baths
3DP	to pH 1.3	3 base baths
K	to pH 1.3	3 base baths with depigmentation
A	to pH 1.3	autolysis
UZ	to pH 1.3	ultrasound, reduced amount of NaOH
0DP2	to pH 0.5	no
1DP2	to pH 0.5	1 base bath
2DP2	to pH 0.5	2 base baths
3DP2	to pH 0.5	3 base baths
K2	to pH 0.5	3 base baths with depigmentation
UZ2	to pH 0.5	ultrasound, reduced amount of NaOH

**Figure A3:**
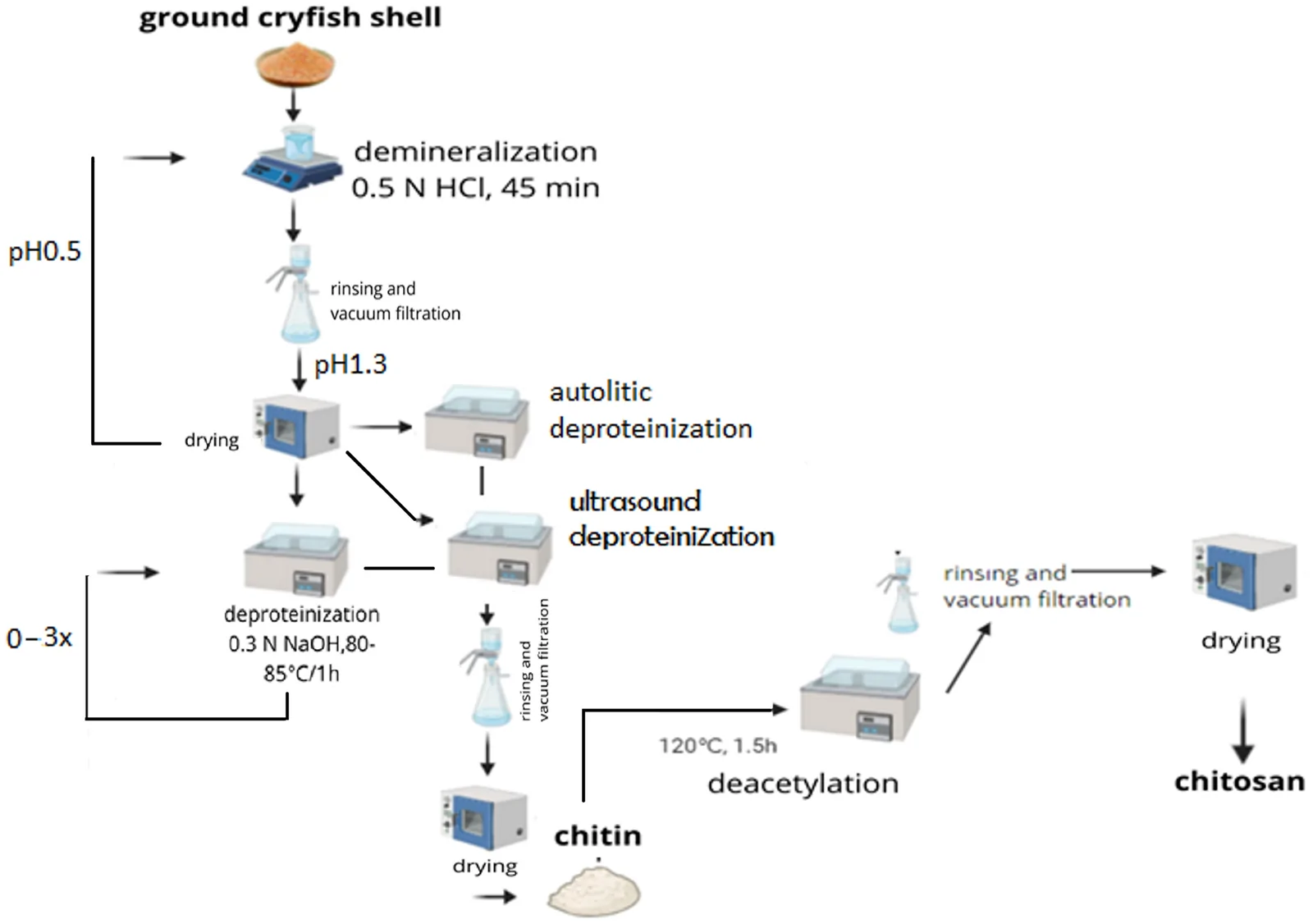
Chitosan extraction flowchart.

## Data Availability

All data generated or analyzed during this study are included in this published article.

## Appendix A

### Appendix A.1

The appearance of the XRD crystallographic peaks of extracted chitosan samples, K as the most crystalline and 0DP2 as the most amorphous, is shown in Figure A1 and Figure A2.

### Appendix A.2

The chitosan extraction flowchart is shown in Figure A3.
